# Reducing blood culture contamination: an environmental imperative

**DOI:** 10.1099/acmi.0.000897.v3

**Published:** 2025-02-26

**Authors:** Sophie Gregg, Niamh Purcell, Maeve Doyle, Grace Chan

**Affiliations:** 1 Department of Clinical Microbiology, University Hospital Galway, Galway, Ireland; 2 Department of Clinical Microbiology, University Hospital Waterford, Waterford, Ireland

**Keywords:** blood culture, bloodstream infection, coagulase-negative staphylococcus, contamination, environment

## Abstract

Blood culture (BC) investigation remains the gold standard for the diagnosis of bloodstream infections. However, BC contamination can have clinical implications for the patient, cost implications for service providers and less well-documented, environmental impacts. Efforts to reduce BC contamination are a long-standing theme in quality improvement initiatives in emergency departments (EDs) and hospitals, prompted by hospital costs, healthcare inefficiencies and antimicrobial stewardship efforts. The WHO’s global analysis of healthcare waste in the context of COVID-19 has reported that tens of thousands of tonnes of extra medical waste were produced from the response to the COVID-19 pandemic, basing its estimates on the quantity of personal protective equipment. Additionally, recent literature has also shown increased BC contamination rates during the COVID-19 pandemic. We performed a retrospective review of the trend of BC contamination during the COVID-19 pandemic in our institution’s ED. We further discuss some of the potential implications of BC contamination, including potential environmental, economic and efficiency implications.

## Data Summary

All the supporting data are provided in the paper. An additional Excel spreadsheet with relevant is avaliable with the online version of this article.

## Introduction

Blood culture (BC) investigation remains the gold standard for the diagnosis of bloodstream infections in febrile illness. BC contamination remains a persistent issue despite revised national guidelines and improved antisepsis. International guidelines recommend that the BC contamination rate should not exceed 3% [1]. BC contamination can lead to a significant clinical burden, with patients often exposed to unnecessary investigations, antimicrobial therapy and lengthened hospital stay [2].

Recent literature has shown increased BC contamination rates during the COVID-19 pandemic [3]. Our study explored the retrieval rate of BC and the trend of BC contamination of the emergency department (ED) in an Irish Tertiary Referral Hospital over a 4.5-year period from January 2018 to June 2022.

The environmental impacts of BC contamination have not been as well elucidated. Previous studies have shown the increase in plastic waste due to the use of personal protective equipment (PPE) during the pandemic and its implications [4]. This study will review the BC contamination trends during the pandemic and discuss the potential environmental, economic and efficiency implications of BC contamination.

## Methodology

A retrospective analysis was performed to evaluate the contamination rates of BC retrieved from patients who attended the ED at University Hospital Waterford (UHW) between January 2018 and August 2022. UHW is a model 4, tertiary referral centre in the southeast of Ireland, with an ED catering for a population of 500 000.

Further data were analysed from three model 3 hospitals to which the South East Regional Microbiology Laboratory at UHW provides laboratory services as a hub and spoke model. The BC contamination rate from these sites was also analysed.

The isolation of coagulase-negative staphylococci (CoNS), a common skin commensal, from BC was used as a proxy for BC contamination[5]. Microbiological data, including the number of BC retrieved, rates of BC positivity and incidence of CoNS skin commensals, were retrieved from the Microbiology Laboratory database.

## Results

**Fig. 1.**
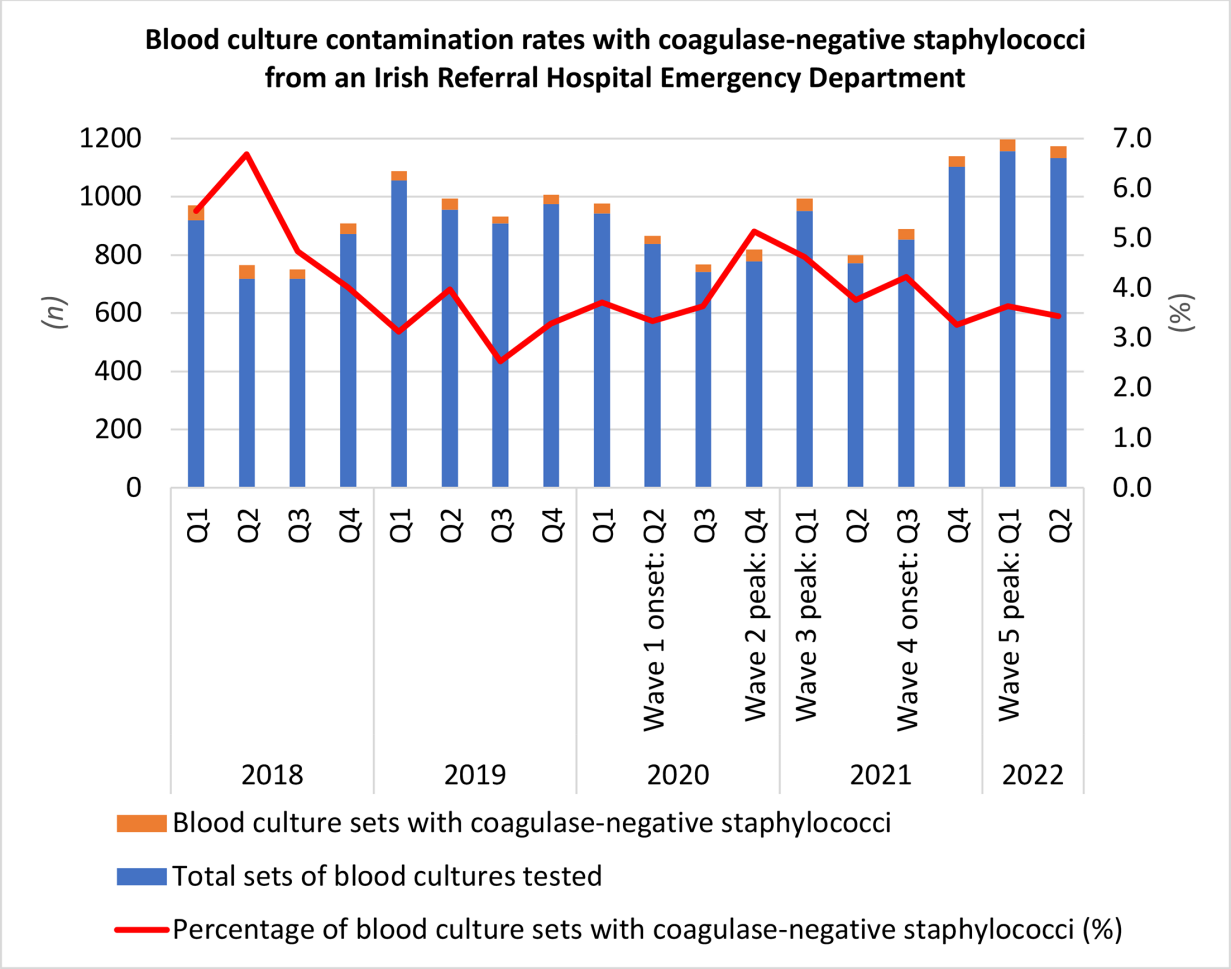
BC contamination rates with CoNS.

A total of 40 247 sets of BC were processed over a 4.5-year period, received from all hospital departments at UHW. Of these, 16 388 sets (40.7%) of BC were taken from ED attendees. Contamination rates over 54 months were analysed to assess the potential impact of the COVID-19 pandemic (summarized in Fig. 1).

In 2018, BC contamination rates were 5.2%, but decreased in 2019, following the implementation of a hospital-wide guideline and proactive education with regard to BC retrieval technique. There were no operational changes during this time period, such as increased staff recruitment, deployment of a cannulation team, or implementation of BC contamination reduction systems. BC retrieval decreased across all quarters during 2020 with the onset of the pandemic compared to 2019. The highest volume of BC retrieval (*n*=1156) was during the onset of the Omicron variant in Q1 of 2022 (BA.1+BA0.2).

The overall total positivity rate of BC (including positive BCs other than CoNS and BCs with CoNS isolated) from the ED was 10.3% over the 54-month period. CoNS were isolated from a range of 3.2% to 5.2% of positive BC samples during the review period (summarized in Table 1). There was a slight seasonal fluctuation in the rate of BC contamination. Despite a relatively low number of BC retrieved, the BC contamination rate was highest (5.1%, *n=*40) during the first winter of the pandemic in Q4 2020 (Alpha variant), followed by a second peak (4.2%, *n=*36) during Q3 2021 (Delta variant).

**Table 1.** Descriptive table of the percentage of total positive BCs and proportion of BC with CoNS isolated

Year	Positive BC	CoNS isolated
2018	12.4% (400/3228)	5.2% (168/3228)
2019	9.3% (362/3894)	3.2% (126/3894
2020	10.2% (335/3298)	3.9% (130/3298)
2021	10% (367/3678)	3.9% (145/3678)
2022	9.7% (223/2290)	3.5% (81/2290)

In 2022, the South East Regional Microbiology Laboratory processed 24 887 sets of BC from the four acute hospital sites. With an estimated contaminant rate of 3.9%, ~971 sets of BC per annum were deemed to be likely contaminants.

## Discussion

BCs are the gold standard diagnostic test for identifying pathogenic organisms responsible for bloodstream infections [5]. Inappropriately taken BCs may lead to the isolation of skin contaminants, such as CoNS. BC contamination rate is a recognized laboratory quality indicator for the pre-analytical stage of BC processing. A landmark study demonstrated that only 12.4% of CoNS isolates were found to be clinically significant [6], yet CoNS account for 44.3% of all positive BC [7]. As CoNS are the most frequently encountered contaminant, they were used as a proxy for contamination in this study.

Prior to the pandemic, the National Sepsis Report 2021 [8] highlighted the need for BC retrieval in febrile illness, and that the crude mortality for patients with COVID-19 infection and concurrent sepsis was more than twice that of those patients without COVID-19 (43.7 vs. 19.6%) [8]. However, a recent meta-analysis demonstrated low bacterial coinfection rates in acute COVID-19 infection (5.6%) with 61.8% of patients with COVID-19 receiving antibiotic therapy [9]. Similarly, in our institution’s retrospective cohort study in Q1 and Q2 of 2021, the majority of hospitalized patients with COVID-19 had BC investigation (70%), and a low rate of bacterial co-infection (5.6%) was observed [10]. Differentiating patients with sepsis due to COVID-19 and those with concurrent bacterial infection proved challenging, demonstrated by high antimicrobial usage during the pandemic period.

Despite the increasing concern of bacterial coinfection, fewer BCs were retrieved at our institution during 2020 (*n*=3298) compared with 2019 (*n*=3894), reflecting a decline in patient ED attendance due to a national lockdown. However, BC contamination rates did not also decrease, likely reflecting ongoing challenges at the point of BC retrieval and the need for continued education and training. In contrast, a number of international studies demonstrated an increased rate of BC contamination for patients with COVID-19 infection and increased BC retrieval [3,11]. The number of BCs retrieved at our institution rose to pre-pandemic levels by the end of 2021. This uptrend coincided with the increased hospital activity typically seen in winter months pre-pandemic, as well as the hospital burden from COVID-19 infections.

Whilst our study did not demonstrate increased BC retrieval or contamination rate during the pandemic, BC contamination rates throughout the course of the pandemic remained above the benchmark of 3% contamination rate. This is likely a reflection of the unprecedented nature of the pandemic coupled with the unresolved resource constraints, overcapacity and understaffing in a level 4 acute tertiary referral hospital. Our ED remained the regional main point of access to healthcare throughout the pandemic. Aside from being a laboratory key performance indicator, BC contamination may signify a bigger crisis masked by the pandemic, i.e. healthcare wastage beyond the laboratory. BC contamination alongside the utilization of single-use plastics, such as PPE, laboratory consumables and clinical and laboratory waste, during the pandemic undoubtedly has had a significant downstream impact.

### Environmental impact

BC contamination leads to increased laboratory resource consumption, generation of laboratory waste and carbon emissions. Laboratories are extremely resource-intensive spaces, using three to six times more energy per surface unit area than office spaces [12]. To prevent BC contamination, single-use plastic instruments and non-recyclable materials are used to prevent the introduction of environmental contaminants. Aside from the laboratory impact, BC contamination can affect hospitals’ wider carbon footprint due to prolonging hospital admissions.

The management of laboratory waste and the disposal of hazardous materials is a considerable cost to healthcare services. Ireland is one of the highest generators of healthcare waste, producing 7.7 kg bed^−1^ day^−1^ compared with 3.3 kg bed^−1^ day^−1^ in the UK [13]. Furthermore, according to the HSE Green Healthcare Programme in 2010, 712 tonnes of cytotoxic anatomical risk waste was produced by laboratories in Ireland and sent abroad for incineration [14]. Although a significant proportion of Irish healthcare risk waste is generated from hospital laboratories (16%), the environmental impact of BC contamination has not yet been fully explored but is likely considerable worldwide.

The importance of providing more sustainable laboratory services has been recognized by the development of organizations such as My Green Lab. Science Foundation Ireland has recently included My Green Lab certification in its Climate Strategy 2024–2027, and the HSE Climate Health Action Strategy 2023–2050 highlights the importance of reducing the environmental impact of our health service, focusing on sustainable building practices, decarbonized transports, sustainable procurement and a waste management framework [15]. The EU has ambitious aims with regard to decarbonization, and as healthcare accounts for 5% of European greenhouse gas emissions, the healthcare sector will be required to make significant contributions towards achieving a net zero-carbon economy [16].

### Economic and efficiency impacts

BC contamination is associated with increased antimicrobial costs, laboratory expenses and costs related to increased length of stay [17]. A case–control study estimated an excess cost of £5001.5 in antimicrobials, laboratory testing, radiological imaging and hospital stays for each false-positive BC result [2]. Furthermore, patients may be exposed to unnecessary antibiotics, particularly intravenous glycopeptides and subsequent therapeutic drug monitoring. One study found that vancomycin administration occurred in 34% of patients with BC contamination [18]. The cost of processing a contaminated BC when compared with a negative BC is increased because of repeated BC retrieval and antimicrobial susceptibility testing.

In 2022, the South East Regional Microbiology Laboratory processed 24 887 sets of BC from the four acute hospital sites, with ~971 BC sets contaminated. As each contaminated BC culminates in an excess hospital cost of an estimated €4404, the financial impact of BC contamination on the hospital group over the course of a year may equate to €4.3 million. By reducing BC contamination by 1% to below the benchmark of 3% (250 BC sets), there would be a significant reduction in regional laboratory processing workload and a potential cost saving of €1.1 million per annum.

Further costs are incurred from the disposal of healthcare risk wastes. At our institution, the disposal of healthcare risk waste by incineration abroad and sterilization at a local plant costs considerably more than general non-risk waste (€2099 per tonne, €1216 per tonne and €142 per tonne, respectively). Through the HSE Green Healthcare Programme, several quality improvement practices in Irish healthcare facilities have been implemented. The segregation of non-risk waste and recyclables from risk waste resulted in a notable reduction of 27 tonnes (8%) per annum in the generation of healthcare risk waste at an Irish hospital with 558 acute inpatient beds, resulting in significant savings for the hospital [19].

We estimated that an average of 1250 kg per annum of laboratory risk waste is generated solely from used BC collection bottles at a single hospital site, which equates to 2.97 kg per acute hospital bed. With 11 892 acute hospital beds in Ireland, this equates to the generation of 35 319 kg of waste by BC bottles alone. This does not include the other day-to-day laboratory consumables used for culture. Although not a high financial cost, this may only be a reflection of the ‘tip of the iceberg’ of laboratory healthcare risk waste production and the downstream impact of waste disposal. Diagnostic stewardship therefore plays a crucial role in mitigating these downstream healthcare wastages.

Regarding human resource consumption, multiple healthcare staff are involved in the retrieving and processing of BC specimens. Clinicians perform clinical assessments at the point of BC retrieval, and ancillary staff, e.g. administrational and logistical staff members, are involved in the pre-analytical stage of specimen processing, followed by medical laboratory scientists who perform analysis of the specimens. Isolation of CoNS can have significant implications with regard to laboratory efficiency. Medical laboratory scientists are required to process the contaminated sample in addition to processing critical specimens, during a time of staff shortage and high laboratory demands as seen during the pandemic. The post-analytical phase involves a Clinical Microbiologist interpreting and communicating the contaminated BC result to clinical teams, which is time-consuming and requires clinical expertise to ascertain the clinical context, explain the relevance of the BC results and advice regarding ongoing patient management. Clinicians are required to review patients in light of BC results, which may include taking more BC and a vicious cycle ensues. Reducing BC contamination rates will disrupt this vicious cycle and in turn will improve the efficiency of healthcare as a whole.

## Conclusions

Our study demonstrated only slight increases in BC contamination during the winter periods of the COVID-19 pandemic, with an overall contamination rate throughout the study period being 3.9%. However, the benchmark for BC contamination is 3%, with lower rates likely achievable. Given the significant clinical, economic and environmental impact, we should be striving to minimize BC contamination. To mitigate the far-reaching impact of BC contamination, improving the preanalytical stage of BC retrieval is essential, with enhanced clinical assessment at the time of retrieval and improved BC techniques. Reducing BC contamination is both a social and an environmental imperative.

Limitations of this study include the fact that our research was a single-site study of a model 4 hospital, assessing samples sent from the ED, where BC contamination rates are higher than in other departments of the hospital. Details regarding the BC-taking technique were not assessed. Our study used CoNS as a proxy for BC contamination, and the isolation of other less common skin contaminants was not included. No patient characteristics, influence of PPE usage, or staffing changes were assessed as part of this study.

## supplementary material

10.1099/acmi.0.000897.v3 Uncited Supplementary Material 1.

## Abbreviations

BC: blood culture
CoNS: coagulase-negative staphylococci
ED: emergency department
PPE: personal protective equipment
UHW: University Hospital Waterford
